# Activation of Xer-recombination at *dif*: structural basis of the FtsKγ–XerD interaction

**DOI:** 10.1038/srep33357

**Published:** 2016-10-06

**Authors:** Andrew N. Keller, Yue Xin, Stephanie Boer, Jonathan Reinhardt, Rachel Baker, Lidia K. Arciszewska, Peter J. Lewis, David J. Sherratt, Jan Löwe, Ian Grainge

**Affiliations:** 1 School of Environmental and Life Sciences, University of Newcastle, University Drive, Callaghan NSW 2308, Australia; 2 Department of Biochemistry, University of Oxford, South Parks Road, Oxford, OX1 3QU, United Kingdom; 3 MRC Laboratory of Molecular Biology, Francis Crick Avenue, Cambridge, CB2 2QH, United Kingdom

## Abstract

Bacterial chromosomes are most often circular DNA molecules. This can produce a topological problem; a genetic crossover from homologous recombination results in dimerization of the chromosome. A chromosome dimer is lethal unless resolved. A site-specific recombination system catalyses this dimer-resolution reaction at the chromosomal site *dif*. In *Escherichia coli*, two tyrosine-family recombinases, XerC and XerD, bind to *dif* and carry out two pairs of sequential strand exchange reactions. However, what makes the reaction unique among site-specific recombination reactions is that the first step, XerD-mediated strand exchange, relies on interaction with the very C-terminus of the FtsK DNA translocase. FtsK is a powerful molecular motor that functions in cell division, co-ordinating division with clearing chromosomal DNA from the site of septation and also acts to position the *dif* sites for recombination. This is a model system for unlinking, separating and segregating large DNA molecules. Here we describe the molecular detail of the interaction between XerD and FtsK that leads to activation of recombination as deduced from a co-crystal structure, biochemical and *in vivo* experiments. FtsKγ interacts with the C-terminal domain of XerD, above a cleft where XerC is thought to bind. We present a model for activation of recombination based on structural data.

The majority of bacterial species have circular DNA genomes. Prior to cell division each circular chromosome must be entirely replicated, unlinked and segregated to ensure that each daughter cell inherits a full genome complement. During or following replication DNA repair processes involving homologous recombination can produce a chromosomal crossover, and any odd number of these events between the circular DNA molecules results in a chromosome dimer^1^. Evidence suggests that this occurs with a probability of 17–40% for each cell cycle^2^,^3^. A chromosome dimer is lethal if unresolved, but most bacteria encode for an efficient site-specific recombination system to convert the chromosome dimers back to monomers, the Xer site-specific recombination system^4^.

Most bacteria encode two tyrosine family recombinases XerC and XerD which bind to the specific chromosomal site *dif* located in the replication terminus region^5^,^6^, although there are notable exceptions where a single Xer protein carries out the reaction^7^,^8^,^9^. The *Escherichia coli dif* site consists of two 11 bp sites that are an imperfect inverted repeat, separated by a 6 bp spacer, or central region^5^. XerC and XerD bind to these sites, with the differences between the repeats imparting specificity to one or other recombinase; XerC binds to one repeat and XerD to the other^10^. Two *dif* sites bound by XerCD can then be synapsed by protein-protein interactions between the two *dif*-XerCD complexes forming a pseudo-tetrameric structure^11^,^12^ with predicted cyclic interactions between monomers as seen in the synapses of other tyrosine recombinases Cre, λ Int and Flp^13^,^14^,^15^,^16^. However, the synapse of (XerCD-*dif*)_2_ is not by itself catalytically competent; it is not until FtsK is present that cleavage by XerD occurs^12^,^17^. After interaction with FtsK, XerD carries out the first pair of strand exchanges to produce a Holliday junction intermediate, that is then resolved by a XerC-mediated pair of strand exchanges to produce a recombinant product^17^.

The FtsK protein is a powerful DNA translocase that is involved in co-ordinating cell division with DNA unlinking and segregation to ensure that the closing septum does not trap or guillotine chromosomal DNA^18^,^19^. FtsK was first identified as a protein involved in cell division^20^ and has been found to interact with several divisome components, leading to the localisation of FtsK to the site of cell division and the recruitment of downstream divisome components^21^,^22^,^23^. The N-terminal domain of FtsK is membrane bound and hexamerises at mid-cell prior to cell division^23^,^24^. The C-terminal domain of FtsK forms a DNA translocase motor, which also hexamerises, and was found to be essential for chromosome dimer resolution through activation of the XerD recombinase^25^,^26^,^27^. Further refinement of the mechanism of activation of XerD showed that it is only the very C-terminal domain of FtsK, called γ, that is absolutely required for recombination^17^. FtsKγ interacts directly with XerD to stimulate its catalytic activity, leading to XerD cutting and exchanging the first pair of strands within a recombinase synapse between *dif* sites^17^.

The FtsKγ domain also plays another role in orienting the FtsK motor to translocate toward *dif* sites; FtsKγ binds specifically to 8 bp sequences termed KOPS that are highly skewed on each chromosome arm to point towards the *dif* site^28^,^29^,^30^. Three FtsKγ domains bind specifically to each KOPS site, which leads to loading and hexamerisation of the FtsK motor domains to one side of the KOPS sequence so that subsequent translocation is always directed towards the *dif* site^31^.

Here we present the structure of the FtsKγ domain interacting with the XerD recombinase and provide biochemical evidence to support the involvement of amino acids at the interaction surfaces in the activation of recombination. A mutant in XerD that disrupts the interaction with FtsKγ is severely impaired for recombination activity on plasmid substrates and is also incapable of supporting chromosome dimer resolution in cells. Further, we demonstrate that a 9 amino acid stretch from FtsKγ that encompasses the interaction surface is sufficient for activation of recombination. It is of note that the same region of FtsKγ that binds KOPS-DNA (between helices 2 and 3) is also responsible for the protein-protein interaction with XerD to activate recombination. Finally, we also provide structure-based models to explain how the FtsKγ-XerD interaction can influence the XerCD-*dif* synapse to promote recombination.

## Results

### Structure reveals the XerD-FtsKγ interaction

**Figure 1:**
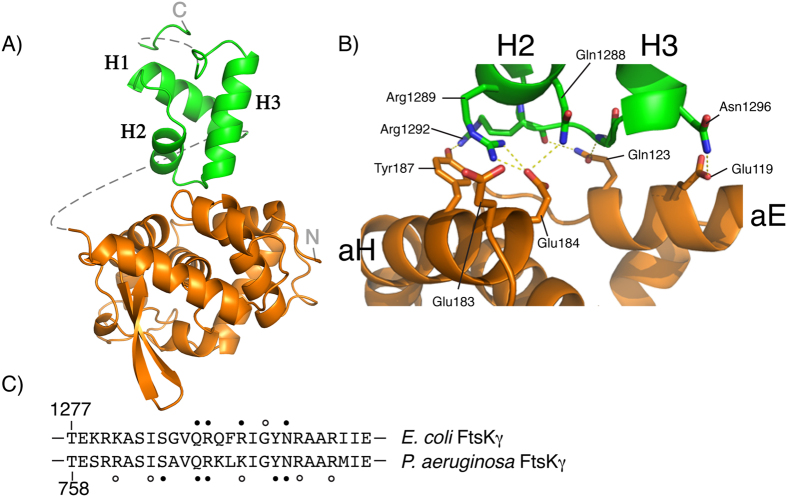
Crystal structure of XerD_C_-FtsKγ interaction. (**A**) The structure of XerD_C_ (orange) interacting with FtsKγ (green). Helices for FtsKγ are marked. Dotted lines show connections for which the electron density was not observed. The N- and C- termini are marked. (**B**) Close up of the interacting amino acids at the interface between XerD_C_ and FtsKγ. Hydrogen bonds are shown as dashed lines. (**C**) Comparison of the amino acids from *E. coli* FtsKγ that interact with XerD_C_ (this study) and the amino acids from *P. aeruginosa* FtsKγ that interact with KOPS DNA^31^. Filled circles above or below the amino acid represent interactions from the amino acid side-chain, and open circles are backbone contacts.

The activation of recombination at *dif* requires the interaction of XerD with the γ domain of FtsK. To understand how this interaction can lead to activation of recombination, a fusion protein between the C-terminal domain of XerD, which contains the catalytic residues required for DNA cleavage and strand exchange, and FtsKγ was produced (XerD_C_–γ) and crystallized. The full length XerD-FtsKγ fusion protein has previously been shown to be a functional, self-activating, recombinase protein at *dif* sites^17^. Attempts to crystallize either XerD with the FtsKγ domain as separate proteins, or to crystallize the full-length XerD-FtsKγ fusion protein both failed to yield high quality crystals. The structure of the XerD_C_-FtsKγ fusion protein was solved by molecular replacement and refined to a resolution of 2.3 Å (Fig. 1), using the previously reported XerD structure (1A0P) and the NMR structure of the *E. coli* FtsKγ domain (2VE8) as search models^30^,^32^. The asymmetric unit contained a single XerD_C_–γ complex, but the flexible linker joining the two protein domains was not visible in the density. Details of the linker sequence and all amino acids for which there is insufficient electron density are shown in supplementary data (Figure S1). The linker is 16 amino acids long, which is of sufficient length that the site of interaction between the XerD and FtsKγ portions of the fusion protein should not be limited. Therefore, the linker may have aided co-crystallisation of the two protein domains but its presence should not interfere with interpretation of the interaction. In addition, biochemical investigations were conducted that confirmed the putative interaction (see below).

Both the XerD_C_ domain and the FtsKγ domain are structurally very similar to the previously determined individual proteins, with no major conformational changes revealed in either protein domain; overall the XerD_C_ domain only varied by an average RMSD of 0.93 Å on matched Cα atoms, while the RMSD between matched Cα atoms for the FtsKγ domain was 0.77 Å. The density observed for FtsKγ was weaker than that for XerD_C_ and increasingly diminished for atoms that were distal from its interaction with XerD, resulting in the final refined model having chain breaks in this region. However, the density for the interface between the two binding partners was sufficient to facilitate an in depth examination of the key molecular contacts (see Figure S2).

The FtsKγ interaction with XerD_C_ is a 563 Å^2^ interface that involves two helices on the surface of XerD_C_ (Fig. 1B). These two helices, αE and αH, are on the opposite face from the putative DNA-binding surface of XerD and the active-site residues^32^. This suggests that the interaction seen in the crystal of XerD with FtsKγ can readily occur whilst XerD is bound to the *dif* site DNA^16^, and activation of XerD by FtsKγ is likely to be allosteric. The interaction site on FtsKγ involves the ends of helices α2 and α3 and the loop that joins them^30^,^31^. It is noteworthy that this is also a region involved in binding of FtsKγ to KOPS DNA, and the amino acids that contact XerD are a subset of those seen to contact DNA (Fig. 1C); not only is FtsKγ a bi-functional domain with protein-protein and protein-DNA interactions, it uses the same surface to carry out both functions. Mechanistically, this implies that FtsKγ cannot interact with KOPS DNA and XerD simultaneously. Further, it may make interpretation of the results of assays using mutations in the FtsKγ domain difficult; recombination efficiency is often used as a readout but determining whether a mutation affects DNA loading of FtsK or XerD activation or both would be problematic.

### Altering residues within the XerD–FtsKγ interface reduces recombination

**Figure 2:**
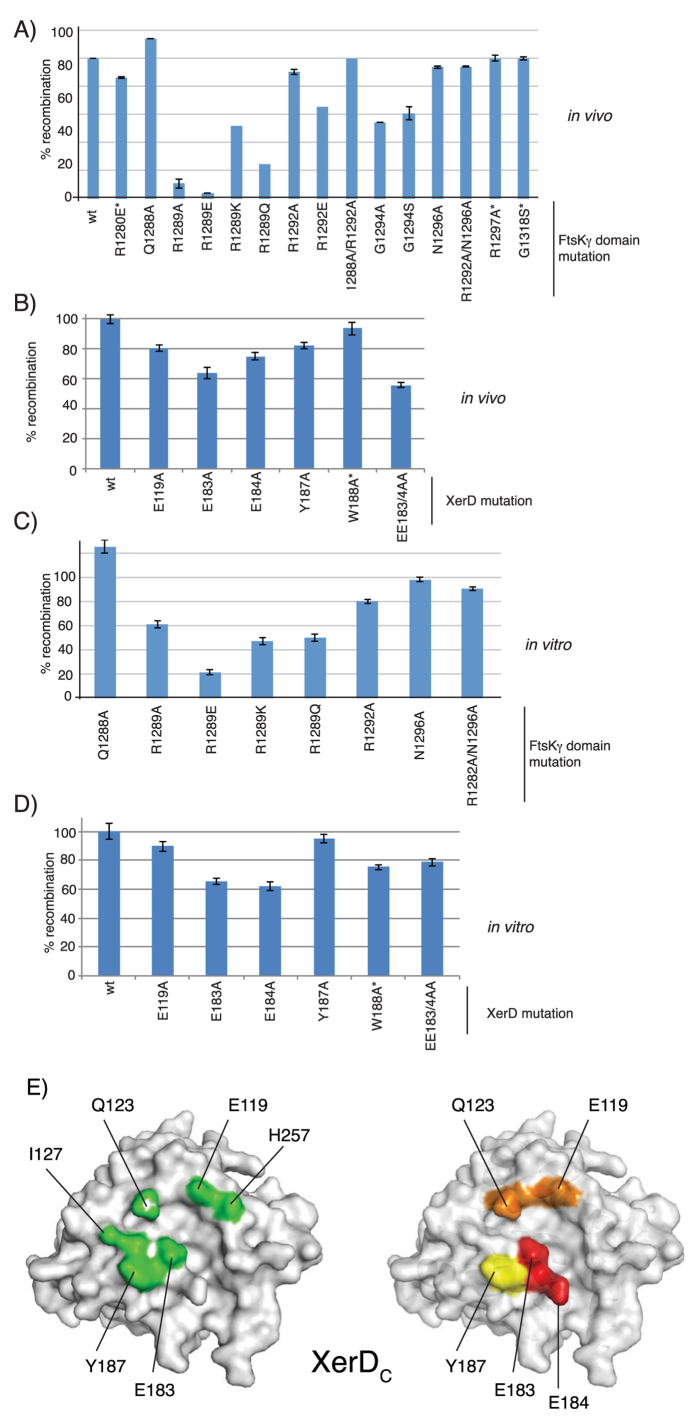
Recombination activity of XerD and FtsKγ interaction mutants is reduced. (**A**) Recombination *in vivo* from XerCγ or XerDγ fusion proteins with mutations as denoted in the FtsKγ domain. Mutations in control residues not seen to be involved in contacts in the crystal structure are denoted with an asterisk (*). (**B**) Recombination *in vivo* from XerDγ fusion proteins with amino acid substitutions in the XerD portion as noted. Recombination in *vitro* using purified fusion proteins from mutations in the *ftsKγ* domain (**C**) or in the *xerD* domain (**D**). (**E**) Two views of XerD represented as space filling models. On the left the amino acids that interact with FtsKγ are highlighted in green and the individual amino acids are labelled. On the right the relative effect upon recombination efficiency of each amino acid mutation described here is colour coded: red (largest effect) through to yellow (smallest effect).

Although the structure of the fusion protein reveals an interaction surface that is consistent with the proposed role of FtsKγ interacting with and activating XerD whilst it is bound to DNA, there was no obvious structural change in XerD that would account for its activation; the active site residues were in the same, inactive, conformation as seen in the XerD structure without γ present^32^. Therefore, it was necessary to confirm whether the observed interactions were indeed responsible for activation of recombination by XerD. The FtsKγ and XerD domains are seen to interact via 6 hydrogen bonds and a salt bridge (Table 1) (Fig. 1B), and the residues responsible for these interactions were targeted for site-directed mutagenesis. Each amino acid was changed to alanine, or other amino acids in addition as noted (Fig. 2). Several other residues on the surface of the FtsKγ domain close to the site of interaction with XerD, but not seen to be directly involved in binding to XerD, were also changed by site-directed mutagenesis: R1280, G1318 and R1297. These mutations should not directly affect the XerD-FtsKγ interaction and were predicted to retain close to wild-type activity, acting as negative controls for the recombination assay. Similarly, the amino acid W188 of XerD, located close to the site of interaction with FtsKγ but not directly involved, was altered. Since the residues of FtsKγ seen to interact with XerD were also involved in contacting DNA during loading of the FtsK motor, it would be impossible to separate the DNA binding function of the residues from activation of XerD. Therefore, to avoid any possible ambiguity in the interpretation of the mutant data, each FtsKγ mutant was produced in a fusion protein with XerC so that loading and translocation of the FtsK motor would be unnecessary, and the XerD was wt. Each XerD mutant was produced in a XerD-γ fusion protein. Both fusion proteins are thought to work by increasing the local concentration of FtsKγ so that the interaction with XerD is more efficient; which recombinase protein the FtsKγ domain is attached to does not affect the reaction.

The resultant XerC-γ or XerD–γ fusion proteins were tested for their recombination activity *in vivo*; each was independently produced in a strain lacking the C-terminus of FtsK where the fusion protein is the only source of FtsKγ. Also present in the strain was a reporter plasmid bearing 2 *dif* sites in direct repeat^17^. In these conditions the “wt” XerC-γ and XerD-γ fusion proteins have previously been shown to be sufficient to efficiently catalyse recombination on the reporter plasmid^17^. Recombination of the reporter plasmid by each mutant was quantified and compared to the relative level seen with the wild-type fusion protein (Fig. 2A,B). Typical gels are also shown in supplementary data (Figure S3). It is clear that several of the amino acid changes at the XerD-FtsK interaction surface reduce the recombination efficiency *in vivo.* The amino acids whose alteration showed the greatest effect on recombination are E183/4 from XerD and R1289 from FtsK. The E184-R1289 pair interact with each other via a salt bridge and hydrogen bonding in the crystal structure (Fig. 1B) that, based on recombination efficiency, appears to be a critical factor in the interaction. Furthermore, consistent with this interaction, mutation to change the arginine of this pair to the oppositely charged glutamate (R1289E) had an even greater negative effect upon recombination, as would be expected from the structure where two negatively charged amino acids would now be juxtaposed. Changes in the FtsKγ residues not observed to interact with XerD in the crystal structure resulted in wild type levels of recombination, confirming the specificity of the interaction (Fig. 2). The relative influence of amino acids on the XerD surface is colour coded in Fig. 2E.

In the structure, it does not appear that XerD E183 is close enough to make meaningful interactions with the FtsKγ domain, and yet its mutation has a large effect on recombination, and the double E183/184 mutant is even more affected than either single mutant. Therefore, E183 must also have a role in the interaction that leads to activation of recombination. The terminal oxygen of E183 is around 5 Å from the guanadinium group nitrogen of R1289, but rotation around the glutamate side-chain carbon linkages would allow a much closer approach between these two residues; it is possible that the role of interaction with R1289 of FtsK is shared between these two glutamates. Alternatively, the mutation of E183 may not affect the interaction with FtsKγ but rather it could affect the interaction with XerC that leads to recombination (see Discussion).

Mutants of each XerC/D-FtsKγ fusion protein were overproduced and purified, as previously described^17^. These proteins were then used for *in vitro* recombination reactions in the presence of XerC and a model plasmid containing two *dif* sites. The relative levels of recombination were again compared to wild-type proteins (Fig. 2C,D). Similar results to those seen *in vivo* were obtained with mutants in the E183/4 pair of XerD and R1289 from FtsK having the greatest effect on recombination.

Since the mutants in XerD for both *in vivo* and *in vitro* recombination were produced in a fusion protein this may have artificially raised the level of recombination seen; the covalent linkage would increase the local concentration of XerD and FtsKγ which could potentially reduce the effect of mutations which lower the affinity of the interaction between the two proteins. Further, *in vivo,* the strain also produced wt XerD from the chromosome and this may have contributed to the background level of recombination, seemingly reducing the effect of each mutation. The fact that a strong effect was still seen for some of the mutants (E184/R1289) shows that these are interactions which contribute greatly to the required association of these two proteins.

Taken together the *in vitro* and *in vivo* recombination data supports the interaction surface seen in the crystal structure being vital for the activation of recombination.

### A minimal FtsKγ peptide sufficient for recombination

**Figure 3:**
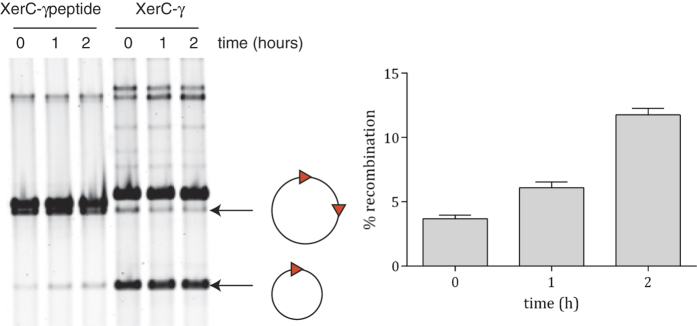
A 9 amino acid stretch of FtsKγ is sufficient to stimulate recombination at *dif*. (**A**) A 2x *dif* reporter plasmid (represented as a circle with two triangles) is in a strain lacking the C-terminus of FtsK. Recombination of the plasmid to delete one *dif* site and produce a smaller DNA circle can occur by expression of either an XerC-FtsKγ fusion (as shown previously^17^) or by expression of XerC-fused to just 9 amino acids from FtsKγ (XerC-γ peptide). Overproduction of the XerC variant was induced at time zero and samples were taken at times indicated. Note that XerC-γ is much more efficient at promoting recombination than the XerC-γ peptide, but expression of both proteins increased recombination over time. (**B**) Quantification of the level of recombination over time for expression of the XerC-γ peptide fusion protein, showing the average of three independent experiments (error bars are SEM).

A fusion protein between XerC and FtsKγ has previously been seen to activate recombination at *dif* as well as, or even more efficiently than, a XerD-FtsKγ fusion (Fig. 1B)^17^. Therefore, in an attempt to confirm that the proposed interactions revealed by the structural data was responsible for the activation of recombination, a fusion protein was generated carrying only 9 amino acids of the FtsKγ subdomain, FtsK residues 1289–1297 (RQFRIGYNR), that contains the FtsKγ interaction surface observed in the crystal structure, attached to the end of a flexible linker at the C-terminus of XerC. This fusion protein was then used in the *in vivo* recombination assay described above. Upon induction a clear increase in recombination was observed, confirming that these few amino acids from the FtsK protein are sufficient to stimulate recombination by XerCD at *dif* (Fig. 3). The 9 amino acid peptide fused to XerC can clearly support recombination. It is perhaps not surprising that it did so far less well than the entire γ domain fused to XerC. Indeed, the XerCγ fusion is sufficiently active that low level production prior to addition of arabinose was enough to recombine the majority of the substrate at time zero, although further recombination was seen over time (Fig. 3A). Expression of XerC alone in this background (i.e. no source of FtsKγ) showed no increase in recombination over the time course (data not shown) as has been seen previously^17^.

### The XerD E183A/E184A mutations do not complement a *xerD* deletion

In order to examine whether the proposed critical amino acids in the FtsKγ-XerD interaction were required for chromosome dimer resolution in growing cells, a co-culture assay was employed^33^. Cells which are phenotypically Xer^−^ show reduced growth compared to a wild-type because chromosome dimer formation leads to cell death in a proportion of these cells^33^,^34^. Two strains, isogenic except that one is *ΔxerD*, were compared for relative growth. A plasmid expressing either wt XerD or a XerD mutant was present in the *xerD* strain to gauge whether the protein could complement the deletion phenotype. Specifically, the XerD E183A, E184A or EE183/4AA mutants were used, as well as a plasmid expressing XerC as a negative control. Note that this assay produced the mutant XerD proteins rather than the XerD-FtsKγ fusion mutants used previously, meaning that chromosomal recombination at *dif* was reliant on the native FtsK protein. Equalised numbers of cells of each strain were inoculated together into the same medium, and were cultured together for ~20 generations. After this period of growth the relative proportion of each strain in the culture was measured by plating onto appropriate selective media. This gives a good readout of how well each XerD protein can complement the *ΔxerD* mutation, and reduce the growth deficiency of Xer^−^ cells.

**Figure 4:**
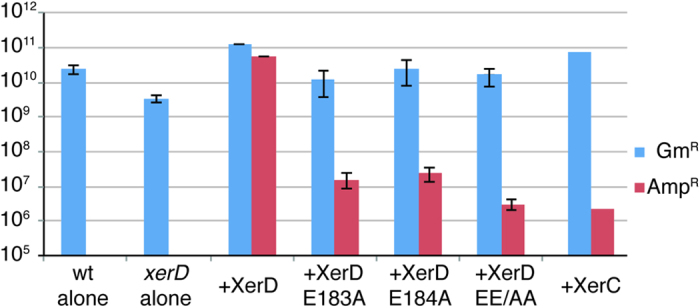
XerD E183A and E184A mutants do not function in chromosome dimer resolution. The graph shows cell counts from a co-culture assay after 20 generations of growth. The two strains used were isogenic and gentamicin resistant (Gm^R^), apart from one having a *xerD* deletion. Growth of the two individual strains separately (with no plasmid) are shown at the left. For co-culture, *xerD* mutant cells carrying the relevant XerD expression vector as noted were also ampicillin resistant (Amp^R^). The ratio of ampicillin resistant cells to gentamicin resistant cells shows the degree of complementation achieved by expression of the XerD variant during co-culture. Results are the average of three independent experiments (error bars are SEM).

The strain expressing wt XerD largely complemented the *xerD* mutation resulting in a relatively minor loss of fitness in this strain compared to wt (Fig. 4). Expression of XerC in this strain did not compensate for the lack of XerD, as expected, and led to a ~10^4^ fold drop in relative cell numbers over 20 generations (Fig. 4). Expression of the XerD E183/4 mutants also largely failed to complement the *xerD* mutation and resulted in a large reduction in fitness of the *xerD* strain relative to the wild-type. As seen previously (Fig. 2) the XerD EE183/4AA double mutant appeared more severely affected than either single mutant and resulted in a reduction in fitness close to that of the XerC negative control. This assay confirms that the identified XerD-FtsK interaction is required for recombination at the chromosomal *dif* site during dimer resolution *in vivo*.

## Discussion

The interaction between the recombinase XerD and the DNA translocase FtsK is vital for chromosome dimer resolution in bacteria^4^,^19^. This interaction leads to activation of the catalytic activity of XerD, with concomitant DNA cleavage and strand exchange as the first stage of the site-specific recombination reaction at *dif*^17^. Structural data presented here show how the interaction between the two proteins occurs; the FtsKγ domain binds on one surface of XerD whilst *dif* DNA would be present on the opposite face of the XerD protein (Fig. 1). Mutagenesis resulting in changes to the amino acids involved in the FtsKγ-XerD interaction confirmed that the observed interactions are required for the activation of the XerD catalytic activity. Further, the presence of just 9 amino acids of the FtsKγ domain that encompasses the XerD-interaction surface were sufficient to stimulate recombination.

A number of studies have used inter-species recombination assays using Xer recombinases and FtsKs from different organisms both for *in vivo* and *in vitro* recombination assays. For example the *P. aeruginosa* FtsK has been seen to activate *E. coli* XerD^26^. This is consistent with the almost perfect conservation of the amino acids in FtsKγ domains from both organisms that contact XerD (see Fig. 1C). Interactions between the Xer and FtsK proteins from *Haemophilus influenzae* and *Lactococcus lactis* have also been examined, and were found to exhibit some degree of species specificity^9^,^35^. The FtsKγ domains from these organisms are reasonably well conserved (Supplementary data, Figure S6) with four of the 5 amino acids involved in contacting XerD in the crystal structure from *E. coli* being the same. However, the interacting residues in XerDs from these organisms are more divergent (Figure S6): of the 5 amino acids found to be important for activation of XerD there are 3 identical and 1 similar in the *H. influenzae* protein whereas the *L. lactis* XerS (there is only a single Xer recombinase in this species) has only 1of the 5 amino acids identical to *E. coli* (Figure S6). Again, this is mirrored in the cross-species activities seen; *E. coli* FtsK can activate *H. influenzae* XerD^35^, but the *E. coli* FtsKγ alone cannot activate XerS from *L. lactis*^9^.

Synapsis between two XerCD-*dif* complexes has been observed in the absence of the FtsKγ domain^11^,^12^. Indeed the data suggest that the synapse adopts a conformation where XerC is inactive and XerD could be active, yet XerD shows no catalytic activity. The addition of FtsK induces a slight conformational change in the synapse that was interpreted as the transition to a XerD-activated (D*) conformation, and leads to formation of the Holliday junction intermediate catalysed by XerD-mediated strand exchanges^12^,^17^. We have shown the molecular detail of the interaction between XerD and FtsK, and that disruption of this interaction leads to the failure to activate XerD. However, there is no obvious conformational change in the XerD protein when it interacts with FtsKγ alone. Therefore, the observed transition of the inactive XerCD-*dif* synapse to the D* state must require interaction with DNA or with XerC, or likely both, in addition to the FtsKγ interaction. Indeed, this makes sense mechanistically; catalysis is only activated within the properly assembled XerCD-*dif* synapse reducing the occurrence of inappropriate XerD-mediated cleavage of the DNA.

**Figure 5:**
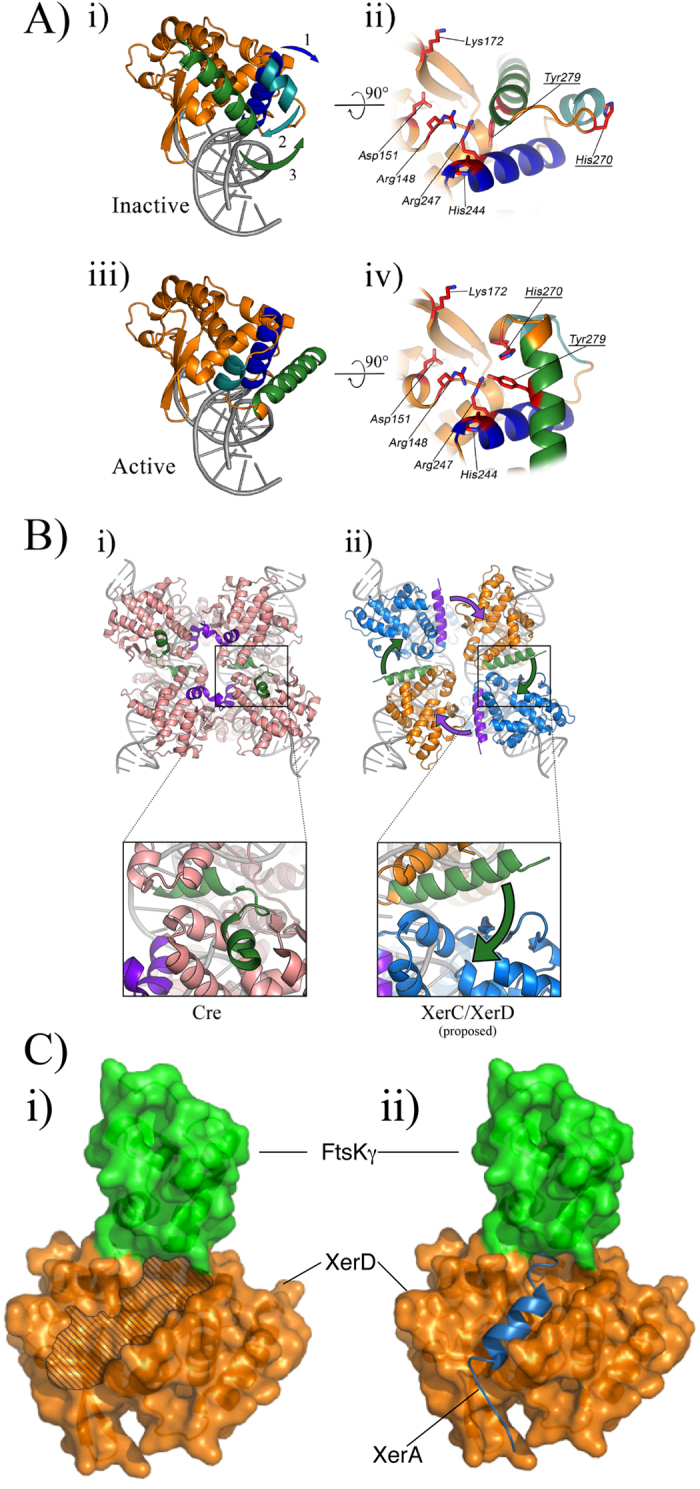
Models of XerD activation. (i) XerD is shown as a ribbon, modelled onto DNA by alignment to the Cre-*loxP* synaptic structure^16^, and (ii) shows a close up of the active site residues in their inactive state, as seen in the XerD_C_-FtsKγ structure. The arrows in (i) indicate movement of helices in order to re-arrange the active site residues to the active configuration as seen in the Cre recombinase and result in the arrangement seen in (iii) and the active site poised for cleavage of DNA (iv). The helices involved in this movement are helix L (blue) helix M (green) and helix N (blue-green). (**B**) (i) The Cre synaptic structure is shown with the very C-terminal helices coloured to emphasise their interaction with the partner recombinase in a cyclic manner. (ii) 4 monomers of the “active” XerD conformation from (**A**) are shown superimposed on the position of the Cre monomers from the synapse in (i). The monomers are coloured to represent XerD (orange) and XerC (blue) in a XerD-active synapse. In order to achieve the same cyclic interactions as seen with Cre the C-terminal N-helices of each XerD monomer must break^36^ and be donated into the adjacent recombinase partner as indicated by the arrows. (**C**) (i) A model representing the “active” arrangement of XerD from (**A**), shown in orange, with the FtsKγ domain in green. The groove in which the C-terminus of XerC is thought to bind is shown by the hatched region and extends to the interaction site of XerD with FtsKγ. (ii) The activated XerD conformation and the XerA structure were overlaid and, the position of the C-terminal tail of XerA is shown (blue helix) occupying the cleft in XerD.

By comparing the XerD_C_–γ active-site structure to that of activated Cre or λ Int, it is clear that two key amino-acids of the XerD active-site are inappropriately positioned for catalysis^13^,^15^: His270 and Tyr279 (Fig. 5A). As previously proposed, a conformational change of the two C-terminal helices of XerD, helix M and N, would produce the active conformation seen in other tyrosine recombinases^36^ (see Fig. 5B). This conformational change would lead to an active site poised for catalysis but would not interfere with the observed XerD-FtsKγ interactions. It is likely that it is this state, where XerD is poised for catalysis but inactive that is acted upon by FtsKγ to activate the catalytic activity of XerD, as proposed by *in vitro* studies^12^.

Another important feature of the Cre and λ Int synapses is the cyclic interaction between monomers obtained by donation of their C-termini to the adjacent monomer^13^,^15^, and these interactions determine which pair of recombinases within a synapse are active and which pair are inactive at any given time. The data from studies on the conformation of XerCD-*dif* synapses along with data that shows that the very C-termini of both XerC and XerD are important for this reciprocal control of partner activation^36^,^37^, suggest that the overall conformation of the synapse closely agrees with those seen for λ Int and Cre^11^,^12^.

By modelling the structure of XerD onto the active structures of Cre or λ Int the XerD M and N helices can be re-arranged to produce an active-site close to those seen for other tyrosine recombinases^36^; this also has the effect of moving the end of helix N to be on the correct side of the molecule to contact the partner recombinase, XerC (Fig. 5B). This movement also removes helix-N from a position where it might sterically hinder the interaction with the C-terminus coming from the partner recombinase. It has also been previously noted that, in order to make the same cyclic contacts as seen with Cre, the N-helix of XerD has to break to reach into the pocket of the partner XerC molecule^36^. This can readily be modelled using the XerD/XerD-FtsKγ-structure superimposed on the Cre-*loxP* synaptic structure (Fig. 5B). There is evidence to suggest this model reflects the physical reality; there is a cleft in the top surface of XerD where the C-terminus of the XerC partner recombinase is proposed to interact (Fig. 5B,C), and amino acids within this cleft have been shown to play an important role in controlling the catalytic activity of the interacting partner recombinase^36^. Further, in the structure of the archaeal Xer homologue, XerA, the very C-terminal helix (helix N) is also seen to occupy this cleft^38^, although in that structure it is folded back *in cis* rather than coming from the partner recombinase. Superimposition of the XerA C-terminus onto the FtsKγ-XerD structure shows XerA extending to a position directly underneath the FtsKγ interaction site (Fig. 5C). The *Pyrococcus abyssi* XerA has a C-terminal tail of similar length to that of XerC from *E. coli* and could reasonably be expected to be a close model (Figure S4). We can, therefore, be confident that this is an important site of interaction between XerC and XerD, and that the FtsKγ domain is positioned to modulate this interaction.

The C-terminal tail of XerC is positively charged with two lysine residues and an arginine among the last 4 amino acids (see Supplementary Fig. 1). The negatively charged E183 from XerD is at the surface, close to the end of the groove where the XerC tail is proposed to occupy, and would be available for charge-based interactions with the C-terminal tail of XerC (see Figure S5). Indeed, mutation that changes E183 greatly reduces recombination at *dif*, and this mutation is synergistic with mutation that alters E184, which directly contacts FtsKγ (Fig. 2). We propose that the reduction in recombination activity from alteration of E183 is a consequence of loss of interaction with the XerC partner recombinase, whereas mutation to change E184 results in loss of interaction with FtsK, and that both these interactions are necessary for efficient activation of XerD-mediated cleavage of *dif* DNA.

**Figure 6:**
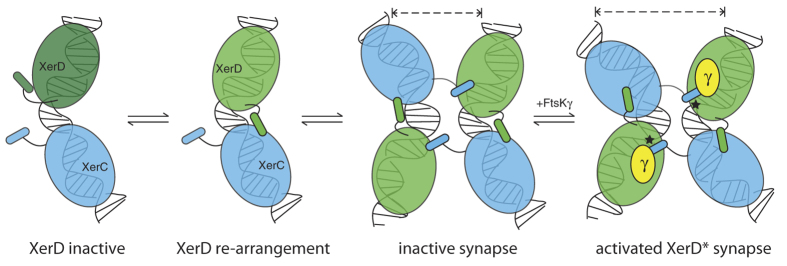
Schematic model of recombination. XerC (blue) and XerD (green) bind to the two halves of the *dif* site. Initially XerD is in the inactive state (dark green) as seen in the crystal structure (Fig. 1). Upon re-modelling of the three C-terminal helices of XerD as described, the active site is now close to the cleavage competent state (depicted by light green XerD) and the very C-terminal helix (helix N) rotates so that it can now interact with the XerC binding partner. At synapsis, two XerCD-*dif* sites come together and the potential for a pseudo-fourfold symmetric arrangement of interactions is present, with the N-helices of XerC stretching across synaptic partners to the neighbouring XerD monomers. Upon interaction of FtsKγ there is a modest re-modelling of the complex to increase the bending of the DNA. The FtsKγ domain interacts above the cleft in XerD in which the XerC N-helix sits. Only when all these conditions are achieved does XerD become catalytically active, as denoted by the black asterisk.

The proximity of the binding site of FtsKγ to the proposed position of the XerC tail in the XerD acceptor cleft could thus be the key to activation of the catalytic activity of XerD. The interaction of XerC, XerD and *dif* is sufficient to produce a synapse with the DNA bent as though XerD would be the active monomer^12^, yet no catalysis is seen without FtsK. A subtle change in the synapse occurs upon interaction with FtsK leading to XerD-mediated strand exchanged to form a Holliday junction (Fig. 6). We propose that the presence of FtsKγ alters the interaction of the very C-terminus of XerC in the acceptor cleft of XerD such that the synapse can now adopt the activated D* conformation^12^. Our model now provides a platform for exciting future experiments to determine how FtsKγ influences XerC-D interaction in the cleft and whether interaction of the very C-terminus of XerC with the FtsKγ domain is required for activation of recombinase activity.

## Methods

### Cloning and mutagenesis

The XerD_C_-FtsKγ fusion protein (XerD residues 111 to 298 followed by a 14 amino acid linker (GGGSEGGGSEGGSG) +2 amino acids (SR) from the linking XbaI restriction enzyme site followed by FtsK residues 1261 to 1329) was amplified from the full length XerD-FtsKγ^17^ using Phusion polymerase and cloned into pBad24 between restriction sites for EcoRI and HindIII. The full amino acid sequence of the fusion protein is shown in supplementary data (Figure S1).

*xerD-ftsKγ* mutants were made by a two-step overlap PCR process, using mutagenic DNA primers and Phusion DNA polymerase (sequences available on request), and cloned into pBAD24. Selected mutant sequences were subcloned into pBAD24-XerD by PCR from the relevant mutant fusion using XerD primers as described previously^17^, or by site-directed mutagenesis using the Q5 Site-Directed Mutagenesis Kit (New England Biolabs). The *xerC–ftsK*γ-peptide fusion sequence was cloned by cutting the pBad24 XerC-FtsKγ fusion^17^ with XbaI and HindIII to remove the FtsKγ domain, and then ligating phosphorylated and annealed oligonucleotides with appropriate overlaps into this vector. All clones produced were verified by sequencing at the Australian Genome Research Facility.

### Protein purification

XerD_C_-FtsKγ fusion overproduction and purification using Ni^2+^ resin was as previously described^17^. Eluted protein was then loaded onto a 1 ml HiTrap heparin HP column (GE Healthcare) in buffer A (25 mM Tris (pH 7.5), 100 mM NaCl, 1 mM EDTA, 1 mM DTT) and eluted with a gradient to buffer B (Buffer A + 1 M NaCl). Protein was then further purified on a 1 ml HiTrap Q HP using the same buffer A and B as above. Eluted protein was concentrated and buffer exchanged into 25 mM Tris-HCl (pH 7.5), 150 mM MgCl_2_, 1 mM DTT, using a VivaSpin 6 centrifugal concentrator (GE Healthcare) to a final concentration of ~9 mg/ml.

All other proteins and fusion proteins for *in vitro* recombination assays were overproduced and purified as previously^17^.

### Crystallization and data collection for XerD_C_-FtsKγ

Initial hits were identified using 200 nl protein drops, using commercial screens (Molecular Dimensions) mixed by Mosquito nano-litre robot (TTP Labtech). Following initial screening, trigonal bipyramidal crystals were obtained by 2μl + 2μl hanging drop vapour diffusion in a solution of 100 mM Bicine (pH9), 10% (w/v) polyethylene glycol (8000 g/mol) over 2–5 days. Crystals were washed in paratone-N and flash frozen in liquid N_2_. Data was collected on MX2 beamline at the Australian Synchrotron, using a wavelength of 0.9184 Å^39^.

#### Structure solution and refinement

The structure was solved by molecular replacement using the known structures of *E. coli* XerD (PDB: 1A0P) and *E. coli* FtsKγ (PDB: 2J5P) as search models (note that only the C-terminus of the XerD structure was used, amino acids 111–298). The program PHASER^40^ placed a single molecule of XerD_C_-FtsKγ in the asymmetric unit. The structure was built using Arp/Warp^41^ and refined using PHENIX^42^ and COOT^43^ (see Table 2 for refinement statistics) to 2.3 Å. 98% of bonds were in the Ramachandran favoured conformation, with 0% outliers.

All structural alignments and structure figures were produced using PyMol (Schrödinger, LLC).

### Recombination assays

*In vivo r*ecombination assays using XerD-γ fusion proteins were carried out as previously published^17^,^30^, but details are given in supplementary material. A similar procedure was used for assessing recombination from XerC-FtsKγ peptide fusions; *E. coli* strain GR51 (AB1157 *xerC ftsK*)^44^, was transformed with pBAD24 derived expression vectors carrying wild type or mutated variants of *xerC-γ* fusion gene along with plasmid resolution reporter, pRB10, a pSC101 derivative (6 kb, SpR) carrying two directly repeated *dif* sites, flanking the KmR gene cassette. pRB10 is almost identical to the pFX142 reporter used previously except that pRB10 lacks the duplication of restriction enzyme sites surrounding the two *dif* sites found in pFX142. Transformants were grown in LB with selection; following 16 h incubations plasmid DNA was recovered from the cultured cells and examined by agarose gel electrophoresis followed by SYBR green staining. Levels of parental and recombinant sized plasmid were quantified using ImageQuant software (GE Healthcare) and percentage recombination calculated.

*In vitro* recombination, using the 2x *dif* reporter plasmid pSI56, was as previously described^17^.

### Co-culture growth competition

Assays were carried out essentially as previously published^17^,^34^,^45^. A 1:1 mixture of the two relevant strains (WX31 (AB1157 *lac*::*tetO*_180_ Gm^R^) and WX31 Δ*xerD* containing the relevant expression vector was prepared and grown in LB at 37 °C to stationary phase (~20 generations)^3^,^17^. The relative abundance of each of the two strains was determined by comparing dilutions plated to select for the XerD expression plasmid (gentamycin + ampicillin), with similar dilutions plated without selection for the plasmid, where both strains will grow (gentamycin alone). The relative colony counts on each plate were determined.

## Additional Information

**Accession codes:** Coordinates and structure factors for the XerD_C_-γ complex have been deposited in the Protein Data Bank under the accession code 5DCF.

**How to cite this article**: Keller, A. N. *et al.* Activation of Xer-recombination at *dif*: structural basis of the FtsKγ–XerD interaction. *Sci. Rep.* **6**, 33357; doi: 10.1038/srep33357 (2016).

## Supplementary Material

Supplementary Information

## Figures and Tables

**Table 1:** Intermolecular contacts between XerD_C_ and FtsK_γ_.

XerD_C_	FtsKγ	Interaction	Distance (Å)
Glu119^Oε1^	Asn1296^Nδ2^	H-Bond	2.93
Glu119	Asn1296	VDW	
Gln123	Arg1292, Ile1293, Gly1294	VDW	
Ile127	Arg1292	VDW	
Glu184^Oε2^	Gln1288^Nε2^, Arg1289^Nη1^, Arg1289^Nη2^	H-Bonds	3.22, 2.53, 2.98
Glu184^Oε2^	Arg1289^Nη1^, Arg1289^Nη2^	Salt-Bridges	2.53, 2.98
Glu184	Gln1288, Arg1289	VDW	
Tyr187_Oη_	Arg1292^Nη1^	H-Bond	2.81
Tyr187	Arg1289, Arg1292,	VDW	
Trp188	Arg1292	VDW	
His257	Asn1296	VDW	

Atomic contacts determined using the CCP4i implementation of *CONTACT*.

Van der Waals interactions defined as non-hydrogen bond contact distances of 4 Å or less.

Hydrogen bond interactions are defined as contact distances of 3.3 Å or less.

Salt-bridge interactions are defined as contact distances of 4.5 Å or less.

**Table 2:** Data collection and refinement statistics (molecular replacement).

	XerD_C_-FtsKγ
**Data collection**	
Space group	P 65
Cell dimensions	
*a*, *b*, *c* (Å)	83.44, 83.44, 88.66
α, β, γ (°)	90, 90, 120
Resolution (Å)	56.02–2.30 (2.38–2.30)*
*R*_merge_	0.12 (1.66)
*R*_pim_	0.04 (0.54)
*CC(1/2)*	1.00 (0.53)
*I*/σ*I*	13.8 (2.1)
Completeness (%)	100 (100)
Redundancy	11.0 (11.2)
**Refinement**	
Resolution (Å)	36.13 - 2.3 (2.38–2.30)
No. reflections	15639 (1566)
*R*_work_/*R*_free_	0.191 (0.287)/0.229 (0.303)
*CC*_*work*_	0.84 (0.65)
*CC*_*free*_	0.85 (0.78)
CC*	0.94 (0.80)
No. atoms	1976
Protein	1915
Ligand/ion	—
Water	61
*B*-factors	Overall 67.5
Protein	67.9
Ligand/ion	—
Water	52.6
R.M.S. deviations	
Bond lengths (Å)	0.01
Bond angles (°)	1.16

Values in parentheses are for highest-resolution shell.
