# Supplementary material for: Activation of Xer-recombination at dif: structural basis of the FtsKγ–XerD interaction
Source: Sci Rep. 2016 Oct 6;6:33357. doi: 10.1038/srep33357 (PMC5052618; doi:10.1038/srep33357)
Supplement: Supplementary Information [file srep33357-s1.pdf]

Activation of Xer-recombination at *dif*: structural basis of the FtsKγ–XerD interaction

Andrew N. Keller[1#], Yue Xin[1#], Stephanie Boer[1], Jonathan Reinhardt[2], Rachel Baker[2], Lidia K. Arciszewska[2], Peter J. Lewis[1], David J. Sherratt[2], Jan Löwe[3], Ian Grainge[1*].

Supplementary material

Methods:

In vivo recombination reaction details.

In vivo recombination reactions using XerD-γ were carried out as previously described [1, 2]. Briefly, the reporter strain consisted of *Escherichia coli* DS9041 ($FtsK_C^-$) transformed with a 2x *dif* reporter plasmid, pFX142, which is based on a pSC101 replication origin and encodes resistance to spectinomycin and kanamycin. *xerD-γ* fusions and variants carrying mutations in the XerD portion were cloned into pBad24 [3]. Individual expression plasmids were transformed in to the reporter strain and selected with ampicillin, spectinomycin and kanamycin. Colonies were grown for 16 hours in LB, without induction with arabinose. Sufficient expression from the P_{BAD} promoter occurs in this time to allow resolution of the majority of the reporter plasmid with "wt" XerD-γ. Plasmid DNA is isolated at this time and electrophoresed in 0.8% agarose in 1x TBE at 55V for 16 hours.

It should be noted that in this system, wt XerD is also expressed from the chromosome and may contribute to higher background levels of recombination using the XerD-γ variant proteins. The recombination percentages shown in Figure 2 for each XerD-γ variant could be artificially high as the FtsKγ domain from the fusion could interact and activate the wt XerD from the chromosome. However, reduction in recombination does imply the XerD variants are compromised in their ability to stimulate recombination.

Table S1: Average B-factors for residues involved in the $XerD_C$:FtsKγ interaction

$XerD_C$ Residue		MC B-factor	SC B-factor	Overall B-factor
119	GLU	46.2	57.5	52.5
123	GLN	43.7	53.0	48.8
127	ILE	42.0	45.7	43.8
184	GLU	38.1	47.6	43.3
187	TYR	37.2	40.0	39.1
188	TRP	34.8	35.9	35.6
257	HIS	47.0	52.2	50.1

FtsKγ Residue		MC B-factor	SC B-factor	Overall B-factor
1288	GLN	93.6	91.8	92.6
1289	ARG	98.8	103.3	101.7
1292	ARG	93.4	103.8	100.1
1293	ILE	89.1	92.7	90.9
1296	ASN	90.8	90.3	90.6

- Calculated using CCP4i implementation of *BAVERAGE*
- MC: Main Chain, SC: Side chain

1 MKDLSEAQVERLLQAPLIDQPLELRDKAMLEVLYATGLRVSELVGLTMSD 50

51 ISLRQGVVRVIGKGNKERLVPLGEEAVYWLETYLEHGRPWLLNGVSIDVL 100

101 FPSQRAQQMTRQTFWHRIKHYAVLAGIDSEKLSPHVLRHAFATHLLNHGA 150

151 DLRVVQMLLGHSDLSTTQIYTHVATERLRQLHQQHHPRAGGGSEGGGSEG 200

201 GSGSRTGAEELDPLFDQAVQFVTEKRKASISGVQRQFRIGYNRAARIIEQ 250

251 MEAQGIVSEQGHNGNREVLAPPPFD 275

Figure S1: Sequence of the construct used for crystallography showing the regions with no electron density. The sequence is colour-coded as in Figure 1, with the XerD$_C$ portion in orange and the FtsKγ domain in green. The linker sequence is shaded grey. Bold black underlines represent amino acids for which the electron density was insufficient and thus are not modelled in the structure in Figure 1.

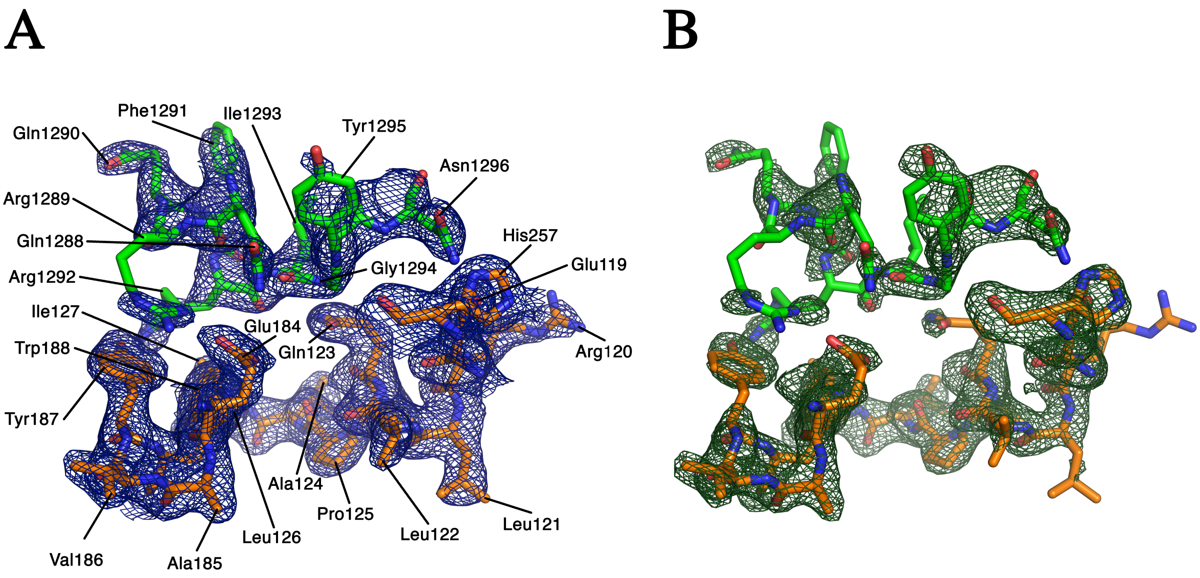

Figure S2: Working and omit maps of the XerD$_C$-FtsKγ interface. A) (2Fo-Fc) map (blue mesh) contoured at 1.0 σ for FtsKγ (green sticks) and 1.3 σ for XerD$_C$ (orange sticks). B) (Fo-Fc) simulated annealing omit map (green mesh) of residues shown in (A) contoured at 2.9 σ.

A)

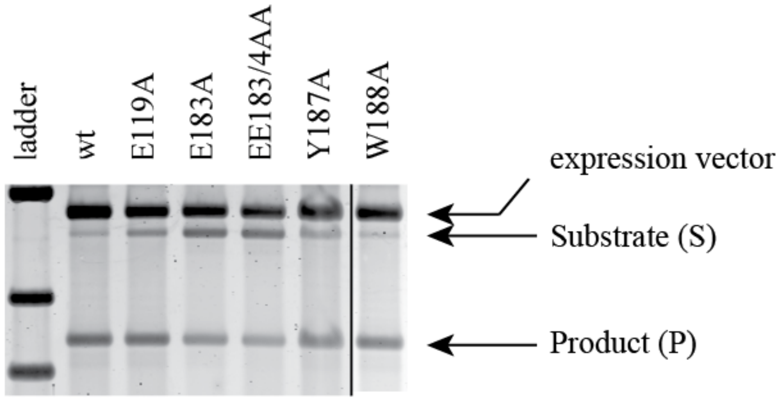

B)

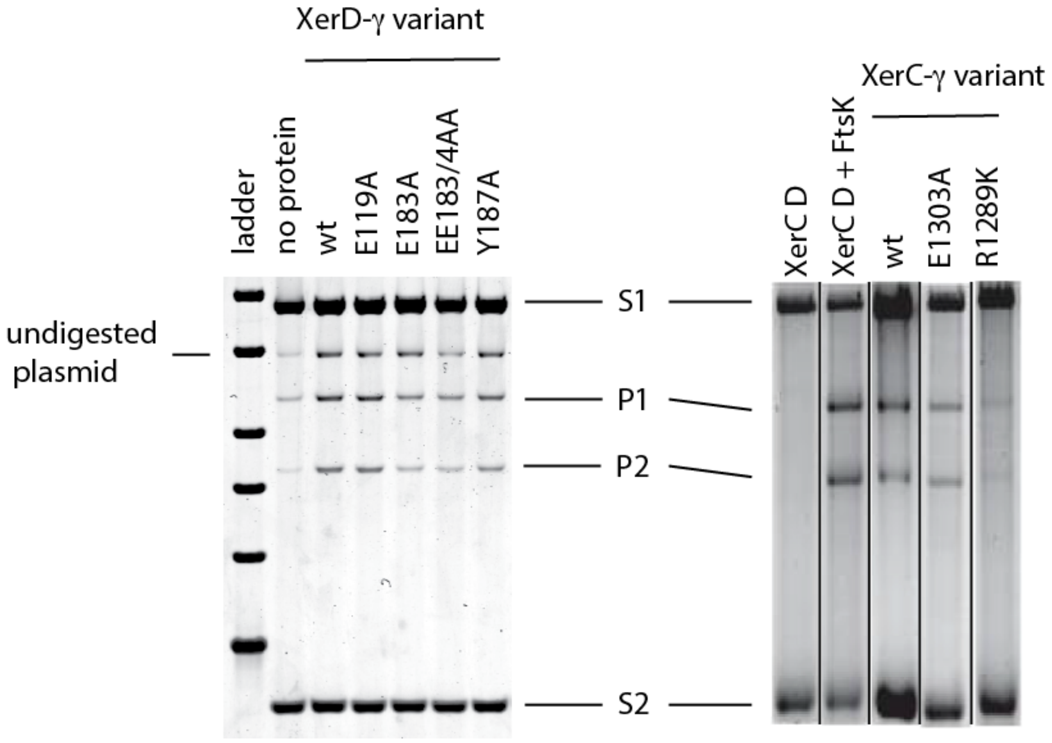

Figure S3. Typical gel images of recombination data shown in Figure 2. A) *in vivo* recombination reactions showing expression of XerD-γ fusion proteins with mutations as indicated. The XerD-γ variants are expressed from the pBad based vector (expression vector). Upon recombination the substrate plasmid (S) is converted to product (P). **B)** *in vitro* recombination reactions. Plasmid was cut with restriction enzyme as described [1] then electrophoresed. Digestion of the parental substrate plasmid yields two bands, S1 and S2, while digestion of the recombination product yields two linear products of different sizes, P1 and P2. The gel on the left also shows some undigested plasmid, and a low background level of recombination that occurred prior to purification of the plasmid substrate.

```
XerD_E.coli        MKQDLARIEQFLDALWLEKNLAENTLNAYRRDLSMMVEWLHHRGLTLATAQSDDLQALLA 60
XerC_E.coli        MTDLHTDVERYLRYLSVERQLSPITLLNYQRQLEAIINFASENGLQSWQQCDVTMVRNFA 60
XerA_P.abyssi      ----MEEREERVRDDTIEEFATYLELEGKSRNTVRMYTYYISKFFEEGHSPTARDALRFL 56
                           *. :    :*.  :   *    *:   :  :   . :             :

XerD_E.coli        ERLEG-GYKATSSARLLSAVRRLFQYLYREKFREDDPSAHLASPKLPQRLPKDLSEAQVE 119
XerC_E.coli        VRSRRKGLGAASLALRLSALRSFFDWLVSQNELKANPAKGVSAPKAPRHLPKNIDVDDMN 120
XerA_P.abyssi      AKLKRKGYSTRSLNLVIQALKAYFKFEG-----LDSEAEKLKTPKMPKTLPKSLTEEEVR 111
                    : .  *  : *    :.*::  *.:          . :  : :** *: ***.:   ::.

XerD_E.coli        RLLQAPLIDQPLELRDKAMLEVLYATGLRVSELVGLTMSDISLRQGVVRVIG-KGNKERL 178
XerC_E.coli        RLLDID-INDPLAVRDRAMLEVMYGAGLRLSELVGLDIKHLDLESGEVWVMG-KGSKERR 178
XerA_P.abyssi      RIINAA-----ETLRDRLILLLLYGAGLRVSELCNLRVEDVNFEYGVIVVRGGKGGKDRV 166
                   *:::         :**: :* ::*.:***:*** .* :..:.:. * : * * **.*:*

XerD_E.coli        VPLGEEAVYWLETYLEHGRPWLLNGVSIDVLFPSQRAQQMTRQTFWHRIKHYAVLAGIDS 238
XerC_E.coli        LPIGRNAVAWIEHWLDLRD---LFGSEDDALFLSKLGKRISARNVQKRFAEWGIKQGLNN 235
XerA_P.abyssi      VPISESLLSEIKRYLESRND---DSPYLFVEMKRKRKDKLSPKTVWRLVKKYGRKAGVEL 223
                   :*:... :  :: :*:        .    . :  :  .::: :.. : . .:.   *::

XerD_E.coli        EKLSPHVLRHAFATHLLNHGADLRVVQMLLGHSDLSTTQIYTHVATERLRQLHQQHHPRA 298
XerC_E.coli        -HVHPHKLRHSFATHMLESSGDLRGVQELLGHANLSTTQIYTHLDFQHLASVYDAAHPRA 294
XerA_P.abyssi      ---TPHQLRHSFATHMLERGIDIRIIQELLGHSNLSTTQIYTKVSTKHLKEAVKKAKLVE 280
                       ** ***:****:*: . *:* :* ****::********::  ::* .  .  :

XerD_E.coli        ------
XerC_E.coli        KRGK-- 298
XerA_P.abyssi      SIIGGS 286
```

Figure S4: Alignment of XerC, XerD and XerA

The sequences of XerC and XerD from *E. coli* and XerA from *Pyrococcus abyssi* were aligned using Clustal Omega [4]. The important catalytic residues, which are conserved throughout the tyrosine-recombinase family, are highlighted by grey boxes. Note the different C-terminal tails of XerC and XerD.

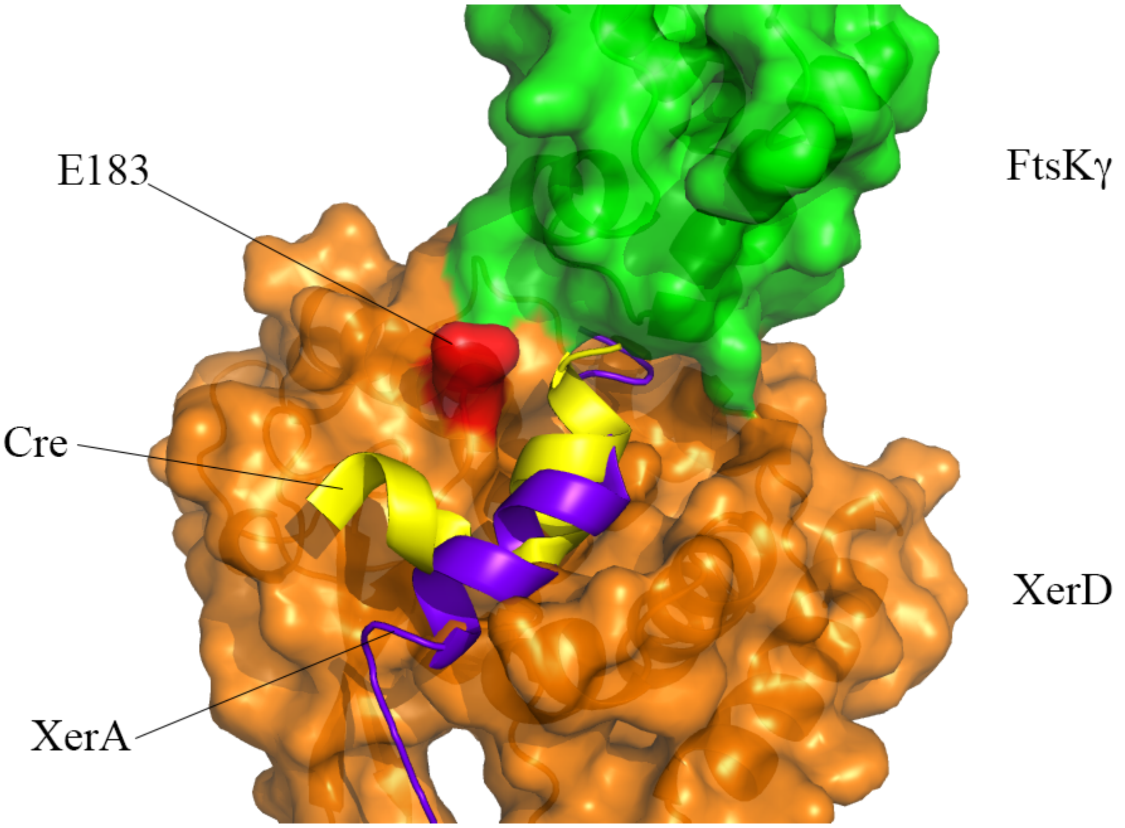

Figure S5: Model showing the C-terminal tails of Cre and XerA occupying the cleft of XerD

The "activated" conformation of XerD is shown in orange with the FtsKγ domain in green. Structural alignments were used to place the C-termini of Cre (yellow) and XerA (blue) onto the corresponding position in XerD. Both occupy the putative cleft where the C-terminus of XerC is expected to interact. The position of the negatively charged E183 is shown, located at the end of the cleft close to the position where FtsKγ sits.

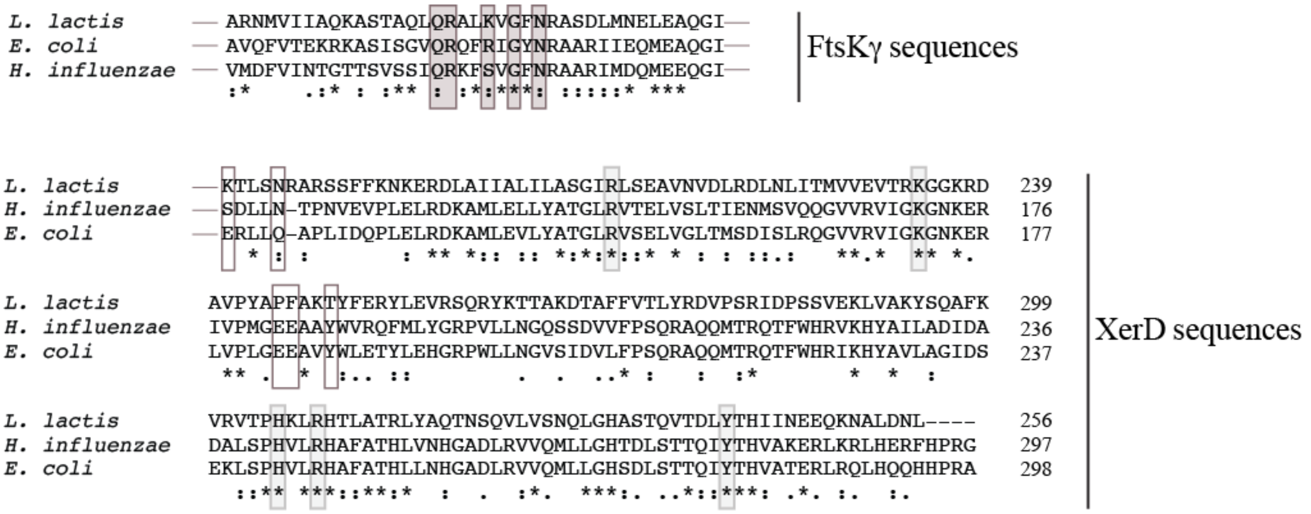

Figure S6: Alignments showing the level of conservation of interacting residues in FtsKγ and XerD across selected species. Top: Alignment of the amino acid sequences of the FtsKγ domains of the indicated organisms in the region where interaction with XerD occurs. Amino acids in grey boxes are the ones observed to interact with XerD in the crystal structure presented here for *E. coli*. **Bottom:** Alignments of the C-terminal portions of XerDs from *E. coli* and *H. influenzae*, and the single Xer recombinase, XerS, from *Lactococcus lactis*. White boxes denote amino acids seen to interact with FtsKγ in the crystal structure from *E. coli*, whereas grey boxes represent the conserved catalytic residues distinctive of the tyrosine recombinase family. Alignments are from Clustal Omega [4].

References:

1. Grainge, I., Lesterlin, C. & Sherratt, D.J. Activation of XerCD-dif recombination by the FtsK DNA translocase. *Nucleic Acids Res* **39**, 5140-8 (2011).
2. Sivanathan, V. et al. KOPS-guided DNA translocation by FtsK safeguards Escherichia coli chromosome segregation. *Mol Microbiol* **71**, 1031-42 (2009).
3. Guzman, L.M., Belin, D., Carson, M.J. & Beckwith, J. Tight regulation, modulation, and high-level expression by vectors containing the arabinose PBAD promoter. *J Bacteriol* **177**, 4121-30 (1995).
4. Sievers, F. et al. Fast, scalable generation of high-quality protein multiple sequence alignments using Clustal Omega. *Mol Syst Biol* **7**, 539 (2011).
